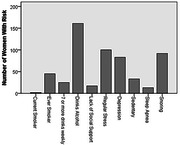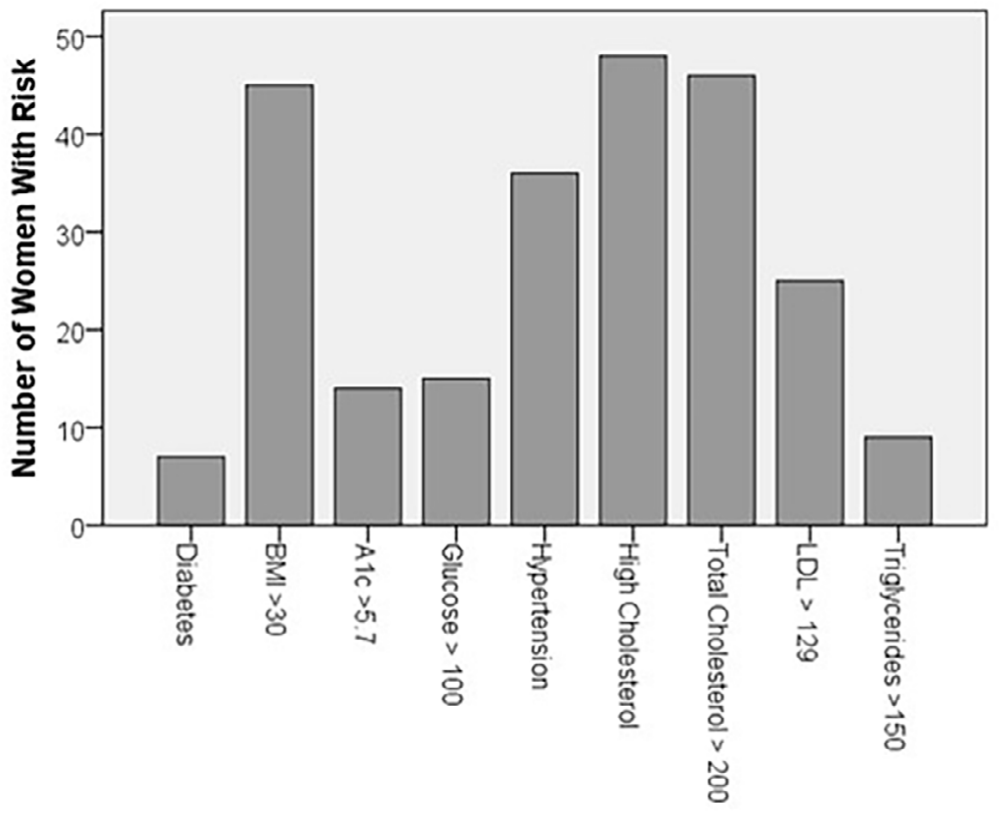# Midlife modifiable AD risk presentation in women across the menopause transition

**DOI:** 10.1002/alz.085727

**Published:** 2025-01-03

**Authors:** Jessica ZK Caldwell

**Affiliations:** ^1^ Cleveland Clinic Lou Ruvo Center for Brain Health, Las Vegas, NV USA

## Abstract

**Background:**

Women are at increased risk for Alzheimer’s disease (AD) compared to men. Given research supporting up to 40% of AD cases as preventable with lifestyle modification, midlife represents a critical time of life to intervene on dementia risks; however, little research has examined women‐specific presentation of risk at midlife, or how menopause staging may impact risk presentation. The aim of this study was to assess dementia risk profiles in women at risk for AD due to family history, including self‐reported and lab‐based modifiable risks, and to determine the role of menopause on risk presentation.

**Method:**

We analyzed baseline data from 207 women patients of a clinical AD risk reduction program (age M = 53.4; education M = 17.0; 88% White; 93% Non‐Hispanic). Total risk score was defined as sum of the following, which were assigned value of 0 (absent) or 1 (present): self‐reported history of diabetes, hypertension, obesity, head injury, sleep apnea, and hearing loss; current regular stress and presence of adequate social support; current and former use of tobacco; drinking more than 7 alcoholic beverages per week; physical and cognitive activity; years of education; and measured BMI>30 (Total possible score = 15). Given drawbacks of self‐report, where available, laboratory values including A1c, fasting glucose, total cholesterol, LDL, and triglycerides were assessed for comparison. Menopause stage was assessed via self‐report (pre‐ or post‐menopause), and for a subset of women, via blood‐based FSH level (pre‐, peri‐, and post‐menopause). Total risk score and lab value differences by menopause group were assessed using Mann‐Whitney and Chi‐square tests.

**Result:**

Most common risks included self‐reported regular stress and depression, and BMI>30 (Fig. 1‐2). Total risks and lab values did not differ by self‐reported (z = ‐0.797) or FSH‐based menopause stage (Chi‐square = 2.3). Qualitatively, elevated lab values and self‐reported symptoms were often more common than self‐reported health conditions.

**Conclusion:**

Total sum of modifiable risks, and lab‐based risk markers did not differ by menopause stage, but rather stress, depression, and BMI emerged as common targetable risks across midlife. Future work should explore whether common symptoms and lab abnormalities shown here in the absence of related diagnoses represent avenues for early screening and intervention.